# *In vivo* comparative study of the effects of using the enamel matrix derivative and/or photobiomodulation on the repair of bone defects

**DOI:** 10.4317/jced.59179

**Published:** 2022-02-01

**Authors:** Valdir-Gouveia Garcia, Valquíria-Simone-Degraf-Gomes Calil, Jânderson-de Medeiros Cardoso, Marcia Hinz, Tiago-Esgalha da Rocha, Edilson Ervolino, Daniela-Maria-Janjacomo Miessi, Luan-Felipe Toro, Daniela-Atili Brandini, Letícia-Helena Theodoro

**Affiliations:** 1 Latin American Institute of Dental Research and Teaching, School of Dentistry Ilapeo, Curitiba, Paraná, Brazil; 2 Group for the Research and Study of Laser in Dentistry, São Paulo State University (UNESP), School of Dentistry, Araçatuba, São Paulo, Brazil; 3 Department of Basic Sciences, School of Dentistry, São Paulo State University (UNESP), Araçatuba, São Paulo, Brazil; 4 Department of Diagnosis and Surgery, School of Dentistry, São Paulo State University (UNESP), Araçatuba, São Paulo, Brazil

## Abstract

**Background:**

The repair of bone defects has been the subject of many studies that have shown inconclusive results as to what is the best bone substitute.

**Material and Methods:**

Bone defects (Ø 2 mm) were induced on the tibia of seventy-two rats, which were distributed into the following four groups/treatments (n=18 each): Control: no treatment; EMD: enamel matrix derived protein; PBM: photobiomodulation therapy (660 nm, 0,035 W, 60 s); EMD + PBM: EMD and immediate treatment with PBM (660 nm, 0,035 W, 60 s). Six animals from each group were euthanized after 10, 30 and 60 days. Histological and immunohistochemistry analyses (osteocalcin - OCN and tartrate-resistant acid phosphatase - TRAP) were performed with scores for each of the biological events.

**Results:**

All performed treatments resulted in an increased filling and maturation of bone tissue, being greater in the EMD and EMD + PBM groups in the 30 day period, compared to the Control group. The immunostaining of OCN was greater at 60 days in all treated groups than in the Control over the same period. TRAP immunostaining was higher at 30 days in all treated groups, and lower in groups EMD and PBM after 60 days, compared to the Control over the same period. There was greater immunostaining in the EMD + PBM group after 10 days than in the Control and EMD groups in the same period.

**Conclusions:**

These results lead to the conclusion that treatments with EMD and PBM, both separate and in association were effective in filling and maturing bone tissue in tibial bone cavities, with greater effectiveness in the period of 30 days in the EMD and EMD + PBM groups.

** Key words:**Enamel matrix proteins, low-level laser, bone, animal research.

## Introduction

The reestablishment of bone defects is one of the great challenges in clinical practice and represents a concern for professionals in decision-making regarding the selection of an appropriate bone substitute. Several options have been presented in the literature, highlighting the use of growth factors and the autogenous bone graft, which is considered the gold standard for bone regeneration. Although the autogenous graft has a high capacity for osseoinduction, some of its disadvantages are the need for a significant amount of material, limited donor areas to obtain the necessary bone volume and rapid resorption. A large number of studies have been dedicated to evaluating other material options to replace autogenous grafts, such as xenogenous bone, allogeneic bone or synthesized materials (1,2).

In addition to these, other biological agents have been reported, such as the concentrated blood derivatives of platelets (platelet-rich plasm, PRP, platelet-rich fibrin, PRF, leukocyte-and platelet rich fibrin, L-PRF, recombinant human platelet-derived growth factor- BB, rhPDGF-BB); proteins (fibroblast growth factor: PGF-2); enamel matrix derived protein (EMD); recombination of DNA (bone morphogenic proteins: BMPs, growth differential factor-5: GDF-5), hormone (3) and photonic therapy (Photobiomodulation, PBM) mediated by low power laser (LPL) or LED (light emitting diode) (4,5).

The EMD was isolated from the Hertwig’s epithelial sheath of porcine dental germ roots, and is commercially called Emdogain® (Straumann, AG, Basel, Switzerland). It represents a set of proteins where amelogenin is the most prevalent, at more than 95% (6). Its biological effects are well documented in the regeneration of periodontal tissues lost due to chronic periodontal disease, both in *in vitro* research (7) as in studies on animals (8) and humans (9), being effective either when applied alone or associated with biomaterial. However, there are studies that have not reported clinical advantages of EMD in the treatment of periodontal infraosseous bone defects (10). Additionally, it is able to reduce or inhibit the differentiation of osteoblasts (11). It is also noteworthy that the mechanism of action of this protein is not yet fully understood (3).

On the other hand, in recent years, the interest of researchers in the use of PBM therapy for bone repair has increased. This photonic therapy, formerly known as low-level laser therapy, has recently undergone a conceptual adjustment, becoming known as photobiomodulation (PBM) therapy, which aims to accelerate the repair, decrease inflammation and reduce pain through irradiation with visible or infrared light. This was due to the fact that biological effects resulting from photonic therapies can occur not only with the use of LPL but also with LED. It is recognized in the literature that LPL-mediated PBM is capable of promoting various biological events like increased mitotic activity, a rise in the number of fibroblasts, collagen synthesis, promoting angiogenesis and the release of growth factors at the injury site, as well as stimulating bone formation and mineralization (12). *In vivo* studies have demonstrated the benefits of PBM in bone repair, either alone or associated with biomaterials (4,13). However, its mechanism of action in bone repair remains inconclusive, since there is a great heterogeneity in the irradiation parameters used.

It has also been observed that the literature lacks studies that have evaluated the effects of EMD and its association with other therapies like PBM on the bone repair of non-periodontal defects. Among the rare reports in the literature there is a randomized controlled clinical study that evaluated this association in periodontal treatment, demonstrating that PBM therapy benefited the effects of EMD in reducing gingival recession, edema and postoperative pain (14). In this study, the authors drew attention to the need for pre-clinical studies capable of evaluating the cellular effects and the biological processes involved. In view of these reports, the purpose of the present study was to evaluate bone repair in bone defects caused in the tibia of rats treated with EMD and PBM, both isolated and in association. The hypothesis of the present study is that the isolated or associated use of these treatments accelerates bone neoformation in bone defects caused in animals.

## Material and Methods

-Animals and Ethics Compliance

Following approval by the Ethics Committee on Animal Use (# 00328-2018, Dentistry School of Araçatuba, São Paulo State University UNESP, Brazil), 72 wistar rats (Rattus norvegicus albinus, Wistar) aged 6 months, weighing 250-300 g were used in this study. The animals were kept in plastic boxes with 4 animals each, in a room with a constant temperature (22 ± 2° C) and controlled light cycle (12-12 h), receiving water *ad libitum* and solid food throughout the experimental period. All procedures were in compliance with the ARRIVE guidelines for animal studies (15).

-Experimental procedures

-Anesthesia and Creation of Bone Defects

After weight assessment, all animals were anesthetized intramuscularly with ketamine hydrochloride (80 mg/kg, Francotar®, Virbac, SP, Brazil) and xylazine hydrochloride (10 mg/kg, Rompum®, Bayer, RS, Brazil). After trichotomy and antisepsis of the internal part of the animal’s legs, a linear incision of approximately 2 cm in length was made, starting immediately below the knee and extended towards the animal’s paw. The tibial bone tissue was exposed and was drilled using a spear-tipped drill (Neodent, Curitiba, Paraná, Brazil), followed by preparation of the bicortical bone cavity with a 2 mm diameter drill (Neodent, Curitiba Paraná, Brazil) mounted on a surgical motor for implantation with controlled speed (980 rpm), under irrigation with saline solution. Immediately after the surgical preparation, the bone cavity was rinsed abundantly with saline to remove the presence of possible bone spicules, leaving the area suitable for the proposed treatments.

-Groups and Treatments

The animals were randomly assigned by a computer-generated Table and distributed into four groups which received the following treatments: Control (n = 18), the cavities remained untreated, filled only with blood clot; EMD (n = 18), the cavities were filled with Emdogain® (Straumann, AG, Basel, Switzerland); PBM (n = 18), the cavities were irradiated by means of an LPL; EMD + PBM (n = 18), the cavities were filled with EMD and then submitted to PBM therapy. In specimens treated with EMD, the protein was slowly applied using a needle into the cavity until it was completely filled. The PBM therapy was performed using a diode laser (InGaAlP, Indium-gallium-aluminum-phosphide) with the following protocol: 660 nm, visible, continuous mode, point-contact, 0.035 W power, spot size 0.0283 cm2 for 60 s at a total energy of 2.1 J, energy density of 74.2 J / cm2 and a power intensity of 1.23 W / cm2, in a single session during surgery. After the treatments were completed, the muscle tissue was re-approximated and stabilized with absorbable sutures, and the dermis / epidermis with non-absorbable sutures.

-Experimental Periods – Euthanasia

At 10, 30 and 60 days postoperative, six animals from each group were euthanized by anesthetic overdose (Tiopental®, Cristália, Itapira, São Paulo, Brazil). The tibiae containing the bone defects were removed, washed and fixed in 4% formaldehyde in 0.1 M phosphate buffer (pH 7.4), for 48 hours.

Laboratory Procedures

-Histological Procedure 

All specimens were identified and underwent demineralization in a solution of ethylenediamine tetraacetic acid - EDTA 10% (Sigma Chemical Co., St Louis, MO, USA) for 30 days. Subsequently, the samples were subjected to conventional laboratory processing and included in paraffin. Semi-serial slides 5 µm thick were obtained in the portion corresponding to the center of the tibial bone defects and collected in silanized glass slides. Some slides were stained with hematoxylin and eosin (H&E) for histological analysis while others were subjected to immunohistochemical processing.

-Immunohistochemical Procedure

The sections selected for immunohistochemical analysis were deparaffinized (xylol) and hydrated in ethanol (100 - 70° GL). Antigenic recovery was performed in a citrate buffer (Spring Bioscience, Pleasanton, CA, USA), in a pressurized chamber (Decloaking chamber®, Biocare Medical, Concord, CA, USA). Next, the slides from each experimental group were separated into two batches, which were incubated with one of the following primary antibodies: anti-osteocalcin (OCN, SC-18319, Santa Cruz Biotechnology®) or anti-tartrate-resistant acid phosphatase (TRAP, SC-30833, Santa Cruz Biotechnology ®).

-Histological Analysis

The semi-quantitative histological analysis was performed by a certified histologist (EE), blinded to the treatments. Semi-quantitative analysis of the histopathological events was performed in a histological section of each animal with reference to the center of tibial bone defects, magnified 50x and 400x. The bone repair pattern was assigned scores, as defined: Score 0 = complete absence of tissue repair; Score 1 = bone defect partially filled with bone trabeculae composed of immature bone tissue; Score 2 = bone defect completely filled by thin trabeculae composed of immature bone tissue; Score 3 = bone defect completely filled with thick trabeculae composed of immature bone tissue; Score 4 = bone defect completely filled with thick trabeculae composed of mature bone tissue; Score 5 = bone defect completely filled with mature compact bone tissue.

-Immunohistochemical Analysis

The immunohistochemical analysis was carried out by a certified histologist (EE) blinded to the treatments. The semi-quantitative analysis was performed at 400x magnification, in a histological section of each animal in all periods.

In the OCN assessment, the following scoring criterion was followed: Score 0 = total absence of immunostaining; Score 1: less than 10% immunoreactive cells and poor marking in the extracellular matrix; Score 2: 10% - 35% immunoreactive cells and poor marking in the extracellular matrix; Score 3: 35% - 65% immunoreactive cells and moderate marking in the extracellular matrix; Score 4: 65% - 90% immunoreactive cells and a strong marking on the extracellular matrix; Score 5: more than 90% immunoreactive cells and strong marking on the extracellular matrix. In the TRAP analysis, the following scoring criterion was applied: Score 0 = total absence of immunostaining; Score 1: less than 2 immunostained cells; Score 2: from 2 - 5 immunostained cells; Score 3: 5 - 9 immunostained cells; Score 4: 9 - 12 immunostained cells; Score 5: >12 immunostained cells.

-Statistical Analysis

The sample size was calculated using software (G * Power, version 3.1.9.2) at α = 0.05 (type I error) and β = 0.8 (type II error), the size of the effect considered average (ES = 0.25). The statistical test MANOVA (Repeated measures between factors) was used, which resulted in 64 animals. Considering losses and complications with the animals 12.5% were added to the group, totaling 72 animals. The data were submitted to statistical analysis using a software (BioEstat - version 5.3, Manaus, Amazonas, Brazil). For immunohistochemical and histological analyzes, the Kruskal-Wallis Analysis of Variance test and the Student-Newman-Keuls post-hoc test were applied. The data are presented in the form of median and interquartile deviations, with a significance of 5% (*P* <0.05).

## Results

-Histological Analysis

**Figure 1:**
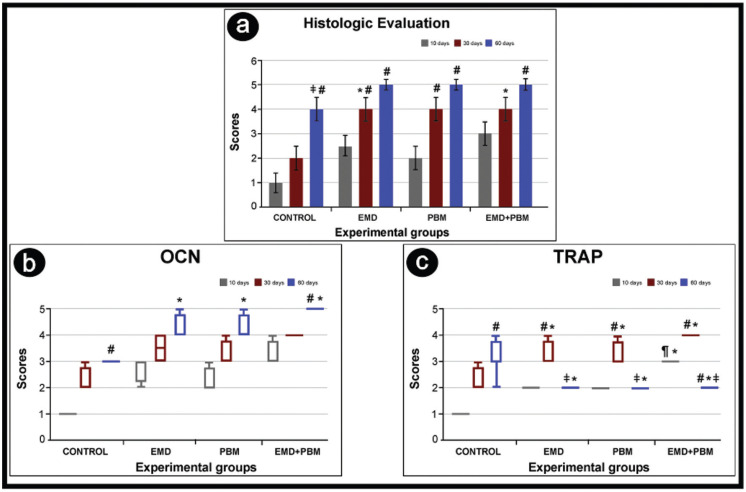
(a) Tissue repair pattern in tibial bone defects (scores), in different experimental groups and periods. Kruskal-Wallis analysis of variance and Student-Newman-Keuls post-hoc test. Symbol: #, statistically significant difference in relation to 10 days in the same group; ‡, statistically significant difference in relation to 30 days in the same group; *, statistically significant difference in relation to the control group, in the same period (*P* <0.05); (b) Graph showing the OCN immunostaining pattern (Scores) in bone defects of tibias, in different experimental groups and periods. Kruskal-Wallis test and Student-Newman-Keuls post-hoc test. Symbols: #, statistically significant difference in relation to the 10 days, in the same group; *, statistically significant difference in relation to the control group, in the same period (*P* <0.05); (c) Graph showing the TRAP immunostaining pattern (Scores) in bone defects of tibias, in different experimental groups and periods. Kruskal-Wallis test and Student-Newman-Keuls post-hoc test (*P* <0.05). Symbols: #, statistically significant difference in relation to 10 days, in the same group; ‡, statistically significant difference in relation to 30 days, in the same group; *, statistically significant difference in relation to the control group, in the same period; ¶, statistically significant difference in relation to the EMD group in the same period.

**Figure 2:**
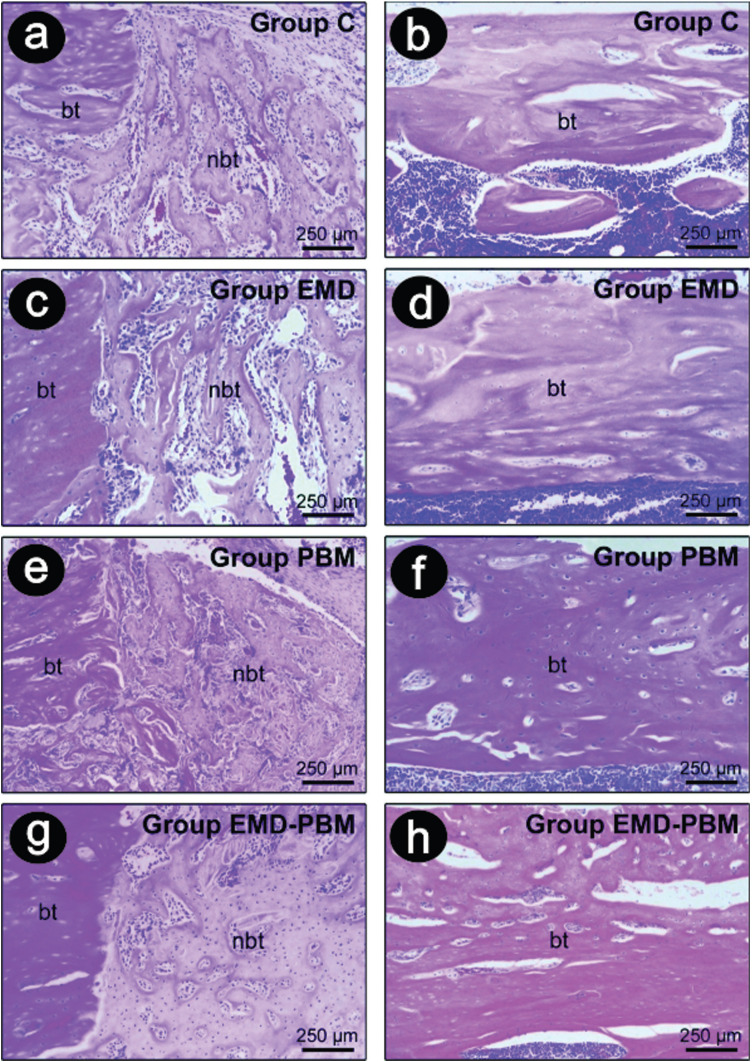
Photomicrographs showing newly formed bone tissue inside the tibial bone defects at 10 days postoperative (a, c , e, g) and 60 days (b, d, f, h) in the Control Group, EMD Group, PBM Group and EMD-PMB Group. Abbreviations: bt, bone tissue original; nbt, new bone tissue. Original magnification: 100x. Scale bars: 250 µm. H&E staining.

Histological analysis revealed that the bone defects were completely filled with thick bone trabeculae composed of mature bone tissue at 60 days in most specimens from groups EMD, PBM and EMD + PBM. In the semi-quantitative intra-group analysis, the Control group presented greater filling and maturation of bone tissue after 60 days than at 10 and 30 days (*P* <0.05). There was greater filling and maturation of bone tissue in groups EMD and PBM in the periods of 30 and 60 days, and in the EMD + PBM group at 60 days, compared with the 10 day period in the same groups (*P* <0.05). In the inter-group semi-quantitative analysis, greater filling and maturation of bone tissue was observed in the groups EMD and EMD + PBM after 30 days than in the Control group (*P* <0.05) over the same period (Figs. 1a,2).

-Immunohistochemical Analysis

**Figure 3:**
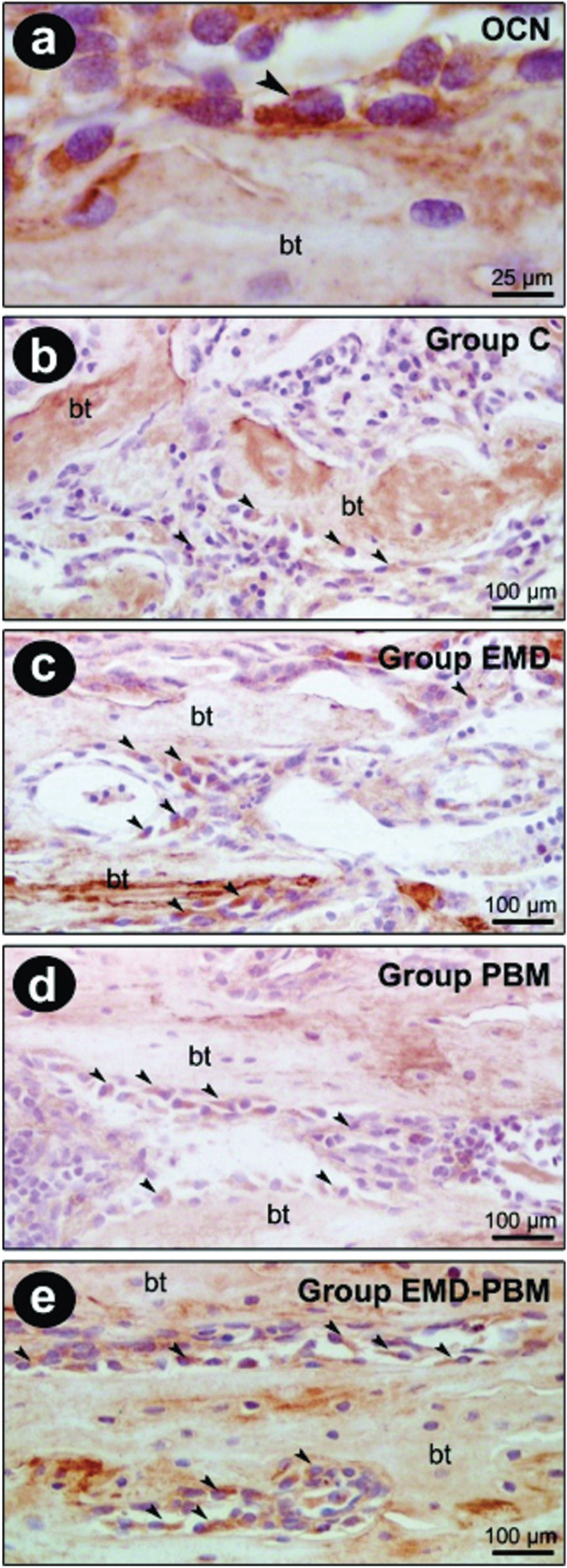
OCN immunolabeling of the surgical defect at 30 days postoperative. OCN-positive cells (a). OCN immunolabeling in bone defects in the Control Group (b), EMD Group (c), PBM Group (d), EMD-PBM Group (e). Abbreviations and symbols: bt, bone tissue; arrows, OCN-positive cells. Original magnification: a, 4000x; b - e, 1000x. Scale bars: a, 25 µm; b - e, 100 µm. Counterstaining: Harris’ hematoxylin.

The employed immunohistochemical technique had a high specificity for the detection of OCN and TRAP. The immunoreactive cells showed a dark brown color confined to the cytoplasm and extracellular matrix in the case of OCN and confined exclusively to the cytoplasm in the case of TRAP. Immunostaining for OCN was evidenced predominantly in osteoblasts, located on the surface of newly formed bone matrix. They were also present in some cells close to osteoblasts, in the bone matrix and in the extracellular matrix of connective tissue in the vicinity of the bone matrix. The intra-group statistical analysis showed that in the Control group at 60 days there was greater OCN immunostaining than at 10 days; a result also observed in group EMD + PBM. In the inter-group analysis, there was greater OCN immunostaining in the EMD, PBM and EMD + PBM groups in the period of 60 days, compared to the Control group in the same period (Figs. 1b,3).

**Figure 4:**
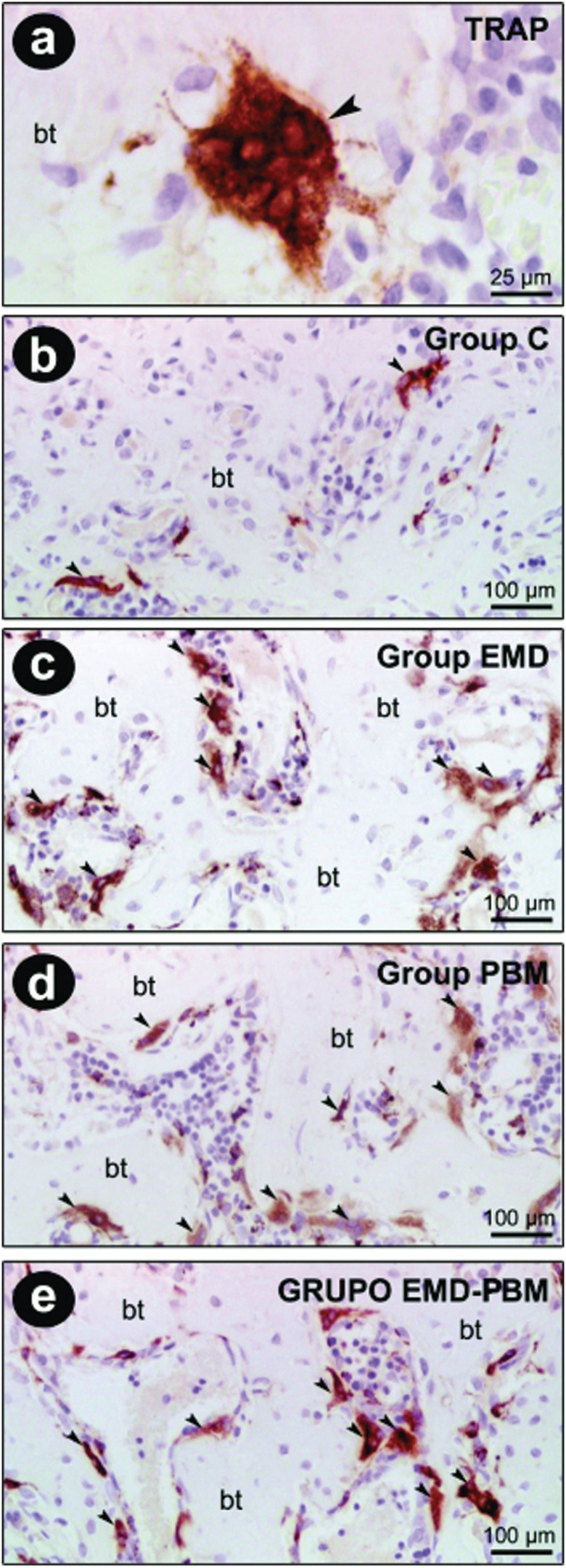
TRAP immunolabeling of the surgical defect at 30 days postoperative. TRAP-positive cells (a). TRAP immunolabeling in bone defects in the Control Group (b), EMD Group (c), PBM Group (d), EMD-PBM Group (e). Abbreviations and symbols: bt, bone tissue; arrows, TRAP-positive cells. Original magnification: a, 4000x; b - e, 1000x. Scale bars: a, 25 µm; b - e, 100 µm. Counterstaining: Harris’ hematoxylin.

TRAP immunostaining occurred predominantly in multinucleated cells present in the vicinity of the bone matrix, i.e. in active osteoclasts. In the intra-group statistical analysis of the TRAP immunostaining it was observed that in the Control group there was a higher number of TRAP positive cells at 60 days than at 10 days (*P* <0.05). Contrarily, in the EMD + PBM group that number was greater at 10 days than at 60 (*P* <0.05). In groups EMD and PBM there was a higher number of TRAP positive cells after 30 days, compared to the period of 10 days (*P* <0.05). The groups EMD, PBM and EMD + PBM showed less TRAP immunostaining at 60 days than at 30. In the inter-group analysis, it was observed that in the EMD, PBM and EMD + PBM groups, there was a greater TRAP immunostaining in the 30-day period, compared to the Control group (*P* <0.05). There was less in groups EMD and PBM at 60 days than in the Control group over the same period (*P* <0.05). However, TRAP immunostaining was higher in group EMD + PBM than in the Control group at 10 days (*P* <0.05). Additionally TRAP was higher in the EMD + PBM group in the period of 10 days, compared to EMD in the same period (*P* <0.05) (Figs. 1c,4).

## Discussion

The present study evaluated the repair process of bone defects surgically created in the tibia of rats that were treated with different agents, being EMD, PBM or both. The use of animals in research is frequent, mainly when it is desired to evaluate the host’s response to different therapeutic modalities and when realization in humans is not viable. For this study rats were used because of their frequent use in research (1,2,4), since they are small in size, easy to obtain and manipulate, low cost, and present an anatomy, systems and biological response similar to that of humans, being the most indicated animals for studies aimed at evaluating bone substitute biomaterials (16).

Bone defects caused in tibia were opted for in this research, because they provide a greater reliability in obtaining results. This model has already been applied in other studies over the years (17), and it does not involve interference from muscle forces in the area. The histological evaluation of the present study showed that all treatments promoted bone formation in all periods, showing defects completely filled by thick bone trabeculae composed of compact and mature bone tissue at 60 days in most specimens from groups EMD, PBM and EMD + PBM.

The results also indicated that the specimens from groups EMD and PBM at 30 and 60 days, and those from the EMD + PBM group at 60 days presented a greater filling and maturation of bone tissue, compared to the 10 day period in the same groups. There was also more filling and maturation of bone tissue in the EMD and EMD + PBM groups in the 30 day period than in the control group over the same period. These results confirm the reports of previous studies that have demonstrated the beneficial effects of EMD (6-8), LPL-mediated PBM (4,5,13) and the association of EMD + PBM (14) on the repair of bone defects. On the other hand, these results call for an adequate professional guidance for decision making in the face of bone defects, because the isolated use of EMD or associated with PBM promotes greater filling and maturation of bone tissue, as evidenced 30 days post-surgery, compared with the control specimens that did not receive any treatment. Reaffirming these histological findings, the immunohistochemical analysis revealed that there were more OCN positive cells in all treated groups at 60 days than in the Control, pointing to a greater differentiation of bone tissue. In addition, specimens from the EMD and PBM groups demonstrated less immunostaining of TRAP positive cells than Control group at 60 days, indicating that the therapies reduced the process of resorption in the long term.

Although with the methodology used in the present study, it was not possible to clarify the action mechanism that resulted in the benefits of these treatments, it is known that both EMD and PBM develop local action and are capable of inducing bone formation (4-9). Studies have shown that EMD is able to promote the release of its main protein amelogenin in a slow and sTable way for a long period, even after a single exposure. Furthermore, it is capable of releasing products that interact with cells, thus stimulating the secretion of growth factors and cytokines in the area (6). Other studies support the hypothesis that EMD can mediate the cellular response to osteoclastogenesis by controlling the transforming growth factor β (TGF-β), blocking osteoclast maturation (via RANKL / OPG) (18) or stimulating the proliferation of pre-osteoblasts, differentiation of osteoblast-like cells and to stimulate the differentiation and proliferation of normal osteoblasts (19). Additionally, it induces the activation of undifferentiated mesenchymal cells, contributing to their osteogenic differentiation (20).

On the other hand, PBM therapy results in the photochemical interaction of light with cellular constituents promoting the acceleration of cell function. It is capable of modulating osteogenesis from the stimulus for the differentiation of bone marrow mesenchymal cells (21) and activating osteoblasts, inducing their proliferation (22) and thus increase the effectiveness of PBM in bone formation (23). This was confirmed in the present study in the groups treated with PBM therapy.

The results of this study lead us to the hypothesis that the EMD protein developed its beneficial biological action in bone repair. The associated use of PBM caused an additional effect, especially in the final periods of research evaluation, where a greater filling of bone defects and maturation of newly formed bone tissue were observed. This observation is reiterated by previous studies which have observed that PBM promotes increased matrix and bone formation (24), osteocalcin, alkaline phosphatase and BMP2 morphogenetic protein (13), in line with our observation of an elevated quantity of OCN positive cells in the treated groups.

It should also be noted that LPL-mediated PBM is able to increase the blood supply in the injury area, through the formation of new blood vessels (angiogenesis) (23). Oxygen plays an important role in all repair phases, due to its ability to contribute to the occurrence of angiogenesis, cell proliferation, bacterial reduction and collagen synthesis (25). Angiogenesis also allows cells and nutrients to reach the affected area, thus favoring oxygen homeostasis, an important condition for tissue proliferation and regeneration to occur (26).

The success of PBM therapy depends on several physical and clinical factors, such as those inherent to the irradiation parameters. It is known that wavelength, energy (J), total energy density (J/cm2) and the frequency of emission are directly related to the biological response. A very low or very high energy density, emission time, the number of applications and power intensity (mW/cm2) may not induce significant biological effects, while excessive light release may cause inhibitory effects (27,28).

The irradiation protocol in the PBM therapy employed in the present study (660 nm, potency 0.035 W, time 60s, 2.1 J, spot size 0.0283 cm2, 2.1 J energy, energy density of 74.2 J/cm2 and 1.23 W/cm2 power intensity) was applied in the trans-surgical area in a timely manner, and selected based on a previous study by our research group that had demonstrated its effectiveness in bone formation.

The wavelength of the laser emitter (660 nm, visible light) was selected due to the fact that the application was performed at the trans-surgical moment, and because visible light interacts with intracellular components of the cells. This promotes action in the cellular respiratory chain, leading to increased cellular energy (adenosine triphosphate - ATP) and, consequently, increased cell function (29). Such action was manifest in the present study in bone defects treated with PBM (groups PBM and EMD + PBM), which were completely filled with thick bone trabeculae and mature bone tissue at 30 and 60 days.

Another important factor was the energy applied to the area of the bone defect. Although energy is directly associated with the power of the emitter and application time, studies have shown that low doses of energy are able to modulate osteoblastic differentiation and proliferation (30), corroborating the results of previous research that used 1 J energy (13), 1.4 J (24), and the 2.1 J employed in the present study, which proved to be adequate for greater bone formation.

On the other hand, studies have shown that a low energy density of 3.15 J/cm2 may not differ with control areas in bone repair, but that energy densities of 31.5 J / cm2, 94.7 J/cm2 and 178 J/cm2 are able to induce faster and more progressive bone healing, shorten initial inflammation, and promote early formation and new bone matrix (24). Therefore, the energy density of 74.2 J / cm2 employed in this study was able to stimulate and accelerate bone formation.

Among the limitations of this study, the presence of blood in the surgical bed prior to the application of EMD stands out, as this may interfere with EMD absorption and its biological effects. It must also be considered that the beneficial effects found in the present study were obtained in animals (rats), and that the translocation of these results to patients should be based on controlled clinical studies. Randomized controlled clinical studies employing LPL-mediated EMD and PBM should be performed with an adequate number of participants to clarify mechanisms and establish treatment protocols for bone repair.

## Conclusions

These results lead to the conclude that treatments with EMD and PBM, both separate and in association were effective in filling and maturing bone tissue in tibial bone cavities, with greater effectiveness in the period of 30 days in the EMD and EMD + PBM groups.
